# Ferroptosis-related gene signature predicts prognosis in kidney renal papillary cell carcinoma

**DOI:** 10.3389/fonc.2022.988867

**Published:** 2022-10-06

**Authors:** Haiying Yin, Mei Lin, Shaoying Liang, Meijuan Wei, Cuiting Huang, Fengfei Qin, Jiejin Nong, Xianchang Zeng, Caimei Nong, Houji Qin

**Affiliations:** ^1^ School of Nursing, Youjiang Medical University for Nationalities, Baise, China; ^2^ Department of Neonatology, Affiliated Hospital of Youjiang Medical University for Nationalities, Baise, China; ^3^ School of Nursing, NingBo College of Health Sciences, Ningbo, China; ^4^ Department of Radiation Oncology, Affiliated Hospital of Youjiang Medical University for Nationalities, Baise, China; ^5^ Department of Renal Diseases, Affiliated Hospital of Youjiang Medical University for Nationalities, Baise, China; ^6^ Department of Infectious Diseases, Affiliated Hospital of Youjiang Medical University for Nationalities, Baise, China; ^7^ Department of Interventional Oncology, Affiliated Hospital of Youjiang Medical University for Nationalities, Baise, China; ^8^ Institute of Immunology, Zhejiang University School of Medicine, Hangzhou, China; ^9^ Nursing Department, Affiliated Hospital of Youjiang Medical University for Nationalities, Baise, China

**Keywords:** ferroptosis, immune landscape, KIRP, immunotherapy, prognostic signature

## Abstract

Ferroptosis, an iron-dependent form of selective cell death, is involved in the development of many cancers. However, the role of ferroptosis-related genes (FRGs) in kidney renal papillary cell carcinoma (KIRP) is unclear. In this study, we examined the mRNA expression profiles and clinical data of patients with KIRP from the TCGA cohort. Consequently, 41 differentially-expressed FRGs were screened using the limma package, and 17 prognostic-related FRGs were identified by survival analysis and univariate Cox regression analyses. Thereafter, a ferroptosis-related gene prognostic index (FRGPI) was constructed based on five FRGs (*AKR1C3*, *SAT1*, *FANCD2*, *HSBP1* and *SQLE*), using lasso Cox and multivariate Cox regression analyses. KIRP patients with high FRGPI scores displayed worse outcomes. Furthermore, the FRGPI was shown to be a reliable independent prognostic factor in both the training and testing cohorts. Comprehensive analysis also showed that the FRGPI can distinguish gene mutation, functional enrichment of immune cells and molecular function-related pathways. Interestingly, low FRGPI score could be more benefit from immune checkpoint inhibitors (ICIs) therapy. Then, the two hub prognostic genes (*AKR1C3 and FANCD2*) as a risk gene for KIRP were identified based on the FRGPI module, and the expression profiles of these two genes were validated using human KIRP cells, besides, we furthermore discovered that *Fancd2* is significantly up-regulated in most cancers and is associated with prognosis. In conclusion, these findings showed that FRGPI can accurately predict the prognosis of patients with KIRP, suggesting that this risk model is a promising prognostic biomarker for these patients. Moreover, targeting ferroptosis (*FANCD2*) could be a potential therapeutic alternative for various cancers.

## Introduction

Renal cell carcinoma (RCC), which makes up 3.7% of all cancers worldwide, is one of the most common genitourinary malignancies (1). Kidney renal papillary cell carcinoma (KIRP) is a subtype of RCC, accounting for approximately 10–20% of RCC cases  (2, 3). A poor prognosis and distant metastases are present at the time of initial diagnosis in about 25–35% of RCC patients (4).. In clinical studies, patients with KIRP showed the second-highest morbidity rate among RCC cases and a poor prognosis (5, 6). Currently, radical or partial nephrectomy remains the mainstream treatment for patients with KIRP; however, the recurrence rate is nearly 40% (3). Researchers have developed therapeutic targets for KIRP, such as cabozantinib, which targets only type 1 KIRP rather than the more aggressive type 2 KIRP (7). Therefore, there is an urgent need to develop a new model to predict the prognosis and potential risk of patients with KIRP to aid in clinical decision-making or explore new therapeutic biomarkers.

Ferroptosis, first proposed by the Stockwell laboratory in 2012, is an iron-dependent type of regulated cell death triggered by the buildup of reactive oxygen species (8). Ferroptosis has intimate ties to many diseases, including neurodegenerative diseases (9), ischemic organ damage (10) and several types of cancers (11). Recently, several studies have confirmed the key role of ferroptosis in tumor development and treatment (12–14). Ferroptosis-related genes (FRGs) such as *P53* (15), Fanconi anemia complementation group D2 (*FANCD2*) (16) and Dipeptidyl peptidase 4 (*DPP4*) (17) play an important role in tumorigenesis and development. Additionally, many tumors have been shown to be sensitive to ferroptosis, including ovarian cancer (18), adrenocortical carcinoma (19), lung adenocarcinoma (20) and hepatocellular carcinoma (21) cells. Besides, ferroptosis cells can regulate anti-cancer immunity by releasing some chemotaxis factors and interacting with natural killer cells, CD8^+^ T cells and other immune cells (22). Therefore, ferroptosis may be a potential target for cancer treatment. However, the part played by FRGs in the emergence and prognosis of KIRP is still not fully understood. In this study, we used The Cancer Genome Atlas (TCGA) to develop a 5-gene ferroptosis-related prognostic index that can be used as a prognostic risk model in KIRP cases to improve patient stratification and facilitate personalized treatment decision-making. Furthermore, the two core prognostic genes (AKR1C3and FANCD2)as a risk gene were identified by the FRGPI and the expression profiles of these two genes in KIRP were confirmed using human KIRP cells. Meanwhile, ferroptosis related gene (FANCD2) may be potential therapeutic targets for a variety of cancers.

## Materials and methods

### Data acquisition and processing

Clinical characteristics and raw RNA-seq data of patients with KIRP, including the expression profiles of paired mRNA, the information regarding the patients’ survival and their clinicopathological features, were acquired from the TCGA database. Subsequently, the RNA-seq data (FPKM values) were converted to transcripts per kilobase million (TPM) values using the limma package (23). The training set consisted of the TCGA dataset, which contained 321 patients randomly split into training (144 patients) and testing groups (142 patients). These groups were analyzed for FRG signatures that could be used to make a prognostic index for KIRP; construct and validate prognostic risk models.

### Somatic mutation and copy number analysis of FRGs

To analyse the mutations and copy number variations (CNVs) of the FRGs in KIRP samples, somatic mutation and CNV information from the TCGA datasets were obtained using TCGAbiolinks (24). The copy number of FRGs and somatic mutations were further analysed using Perl and the R package of maptools (25). The differential expression of FRGs between the normal and KIRP samples was analysed using the Wilcoxon test with the ‘limma’ package. *p<* 0.05 was considered the screening threshold. Pearson correlation algorithm in the R software was used to analyse the correlation between the FRGs and the occurrence and development of KIRP.

### Construction of protein–protein interaction (PPI) network and pathway enrichment analysis

The PPI network of FRGs was constructed using the STRING database (https://www.string-db.org/, version 11.5) and gene interactions with combined scores ≥ 0.4 were selected to build the PPI network. The Gene Ontology (GO) and Kyoto Encyclopedia of Genes and Genomes (KEGG) of the significantly different FRGs (*p*< 0.05) were constructed utilizing ‘clusterProfiler’ in the R package (26).

### Unsupervised clustering of established FRGs

To study the biological properties of differentially-expressed FRGs in patients with KIRP, we used the ‘consensusclusterplus’ package and repeated the steps 1000 times to confirm the classification’s stability (27). Subsequently, using the ‘survival’ package, the Kaplan-Meier curve was used to depict the survival differences between different clusters.

### Gene set variation analysis and exploration of tumor-infiltrating immune cells

‘GSVA’ R package was used to perform GSVA (28) to explore the differences in the pathways and biological processes associated with the FRGs between the dataset samples. The gene set of ‘C2. Cp.kegg. V6.2. Symbols’ was obtained from the MSigDB database to run GSVA. *p*< 0.05 was considered statistically significant. To delve deeper into the landscape of immune infiltration between distinct clusters, a single-sample gene set enrichment analysis (ssGSEA) (29) was performed to calculate the infiltration levels of 23 different types of immune cells.

### Construction of the FRG signatures in KIRP

A survival analysis was performed to evaluate the prognostic value of the FRGs in KIRP patients, and geprognosticnes with *p<* 0.05 were selected as prognosis-related genes. Further, univariate Cox regression analyses were run to determine the association between FRG expression and clinical prognosis. Then, these prognostic genes were optimized by lasso Cox analysis based on “glmnet” package. The independent prognostic geneswere afterwards identified to construct the FRGPI by multivariate Cox analysis based on these optimize prognosis-related genes. Specifically, the expression value of each FRGs was multiplied by their associated coefficient; the FRGPI in KIRP was constructed as follows:




FRGPI(riskscore) = ∑ = 1ncoef (FRGs) * expr(FRGs)

FRGPI is a prognostic risk score for patients with KIRP. These patients were split into high-risk (risk^hi^) and low-risk (risk^lo^) groups in accordance with the median value of FRGPI. The Kaplan–Meier method was performed to determine the prognostic value of the FRGPI based on the median FRGPI score, and univariate and multivariate Cox regression analyses were carried out to determine the independence of FRGPI against other clinicopathological features. The receiver operating characteristic (ROC) curve and area under the curve (AUC) was used to evaluate the accuracy of FRGPI’s predictive capabilities.

### Comprehensive analysis of molecular and immune characteristics and immune therapy in different FRGPI subgroups

To identify differences in signalling pathways of differentially-expressed FRGs between the risk^hi^ and risk^lo^ groups in KIRP, the limma and cluster Profiler package of R was used based on the gene set enrichment analysis (GSEA) method *(p*< 0.05 and FDR< 0.25). Information on gene mutation data of patients with KIRP was obtained from the TCGA data portal (https://portal.gdc.cancer.gov/) and the quantity and quality of gene mutations in the risk^hi^ and risk^lo^ groups were analysed using the maftools R package (25). Subsequently, the ssGSEA package (30) was used to explore the FRG expression levels in tumour-infiltrating immune cells in the different FRGPI subgroups. We also evaluated the differences in responses of patients with KIRP to anti-PD-L1 and CTLA-4 immunotherapy between the two FRGPI subgroups.

### Effects of ICIs treatment between high-and low-FRGPI group

To further investigate the effects of ICIs treatment in different FRGPI subgroups for KIRP, Tumour Immune Dysfunction and Exclusion (TIDE) algorithm was carried out to determine the ICI response of distinct FRGPI patterns in KIRP patients (31).

### Cell culture

CaKi-2 KIRP cells, CKRC-39 cells and HK-2 cells (normal human renal cells) were purchased from the Vinhaket corporation (Shanghai,China)(a list of cell lines was provided in **Supplementary Table S1**). CaKi-2 cells, CKRC-39 cellsand HK-2 were cultured in DMEM supplemented with 10% FBS, and were maintained in a humidified incubator at 37°C with 5% CO_2_-95% air.

### RNA isolation and quantative PCR

Total RNA was extracted with TRIzol reagent (TaKaRa, Kusatsu, Shiga, Japan) and reverse transcribed into cDNA using a cDNA synthesis kit (Toyobo, Osaka, Japan), and a SYBR Green Master Mix kit (Vazyme, Q221-01) was carried out to perform qPCR on the Roche LightCycler^®^ 480II platform (Roche Diagnostics, USA). The expression of the mRNAs of interest was normalized to GAPDH for cell. The primers used for the qPCR were listed in **Supplementary Table S2**. Data were analyzed by the 2^-ΔΔCt^ method.

### Western blotting

For western blotting, total cells were washed with ice-cold PBS, lysed in SDS buffer on ice and boiled for 10 min at 100°C. Then, proteins in the samples were separated by SDS–PAGE, transferred onto PVDF membranes (Millipore) and probed with the corresponding primary antibodies and horseradish peroxidase (HRP)-conjugated secondary antibodies. An ECL Kit (MultiSciences, Hangzhou, Zhejiang, China) was used to visualize the bands.

### Statistical analysis

Differential gene expression analysis was performed using limma R package. Correlation analysis was conducted using the Spearman method. The statistical difference between the two groups was calculated using the Wilcoxon rank sum test, while the Kruskal–Wallis test was used to compare more than two groups. All statistical analyses were carried out using R software (version 4.1.2).

## Results

### Genetic landscape of ferroptosis in KIRP

**Figure 1:**
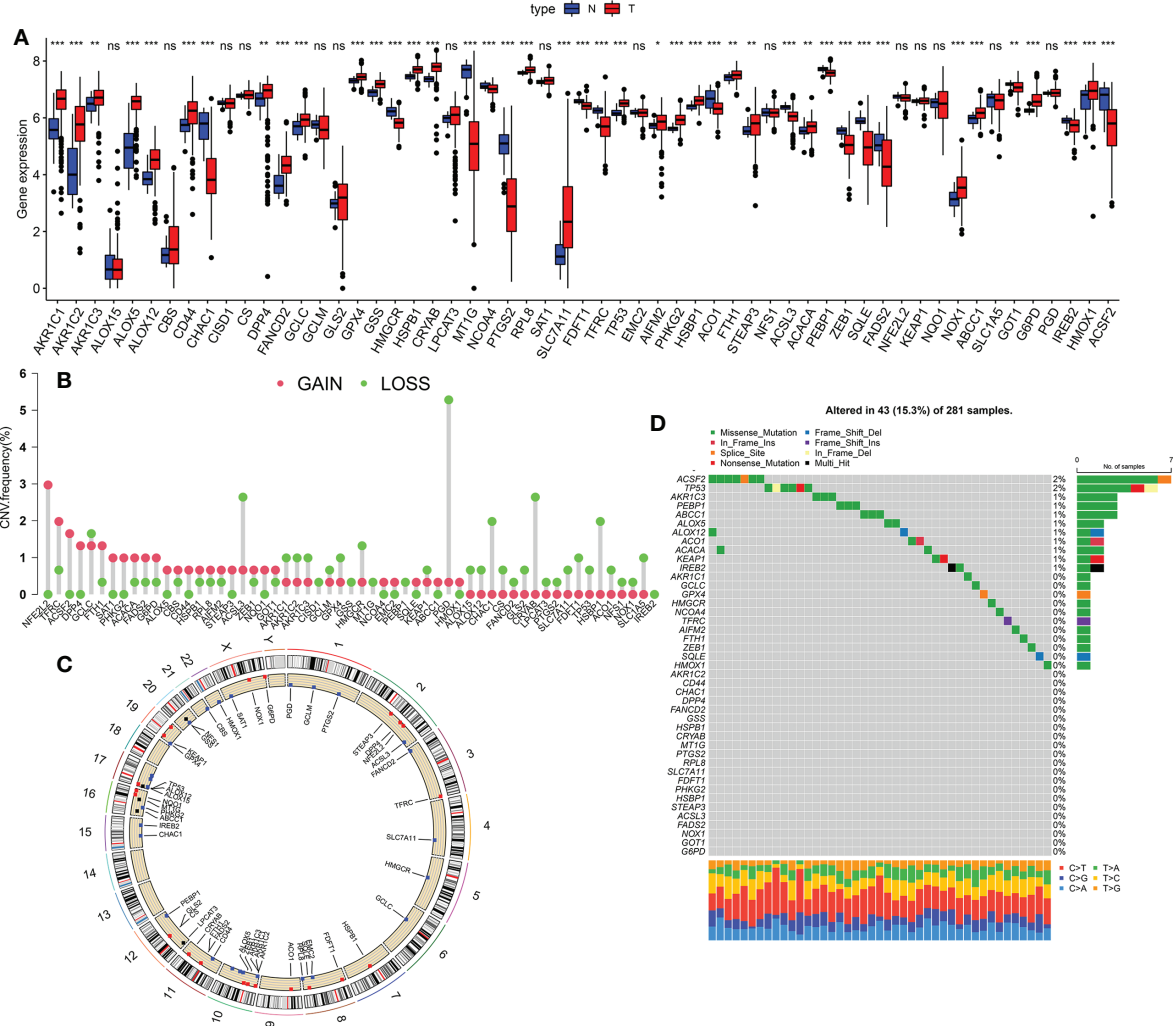
Genetic landscape of ferroptosis in KIRP. **(A)** The expression of FRGs between normal tissues and KIRP tissues. Red, tumour sample; blue, normal sample. **(B)** The CNV frequency of m6A regulators in the TCGA cohort. Red represents an increase in the copy number and green represents the loss of copy number. **(C)** Circle graph of the specific location of the FRG. **(D)** The somatic mutation frequency of FRGs in patients with KIRP in the TCGA dataset *p < 0.05; **p < 0.01; ***p < 0.001; n.s., not significant.

Using the TCGA KIRP dataset, 41 differentially-expressed FRGs were screened between KIRP and normal lung tissues, including 26 upregulated and 15 downregulated genes (**Figure 1A**). We further explored the incidence of copy number mutations in the 41 FRGs. More than half of the FRGs showed copy number amplification, while the deletion copy of *ACSP9*, *GPX4*, *NLRP7*, *IL18*, *ELANE*, *NLRP6*, *PLCG1*, *CASP6*, *CASP3*, *NLRP1* and *PRKACA* were examined (**Figure 1B**); **Figure 1C** showed that the chromosomal position of the CNV mutation in the differentially-expressed FRGs. We also explored somatic mutations of the differentially-expressed FRGs; the genes *ACSF2* and *TP53* showed the highest mutation frequency, followed by *AKR1C3*, *PEBP1*, *ABCC1*, *ALOX5*, *ALOX12*, *ACO1*, *ACACA* and *KEAP1* (**Figure 1D**).

### PPI network and functional enrichment analysis of differentially-expressed FRGs

The interactions of the 41 differentially-expressed FRGs were investigated using PPI network analysis, and the results are shown in **Supplementary Figure S1A**; the correlation network of these genes is displayed in **Supplementary Figure S1B**. To learn more about how the biological functions and pathways of the differently expressed FRGs, GO and KEGG analysis of these genes was carried out. GO analysis revealed that most differentially-expressed FRGs were involved in response to oxidative stress, cellular response to chemical stress and cellular response to oxidative stress; organelle outer membrane, outer membrane and lamellipodium membrane; oxidoreductase activity, carboxylic acid binding and monooxygenase activity (**Supplementary Figure S1C**; detailed results of the GO enrichment analysis are provided in **Supplementary Table S3**). Moreover, KEGG pathway analysis suggested that these genes were markedly enriched in Ferroptosis, Glutathione metabolism, Arachidonic acid metabolism, microRNAs in cancer, Fluid shear stress and atherosclerosis (**Supplementary Figure S1D**; detailed results of the KEGG analysis are provided in **Supplementary Table S4**).

### Tumor classification based on the differentially-expressed FRGs

**Figure 2:**
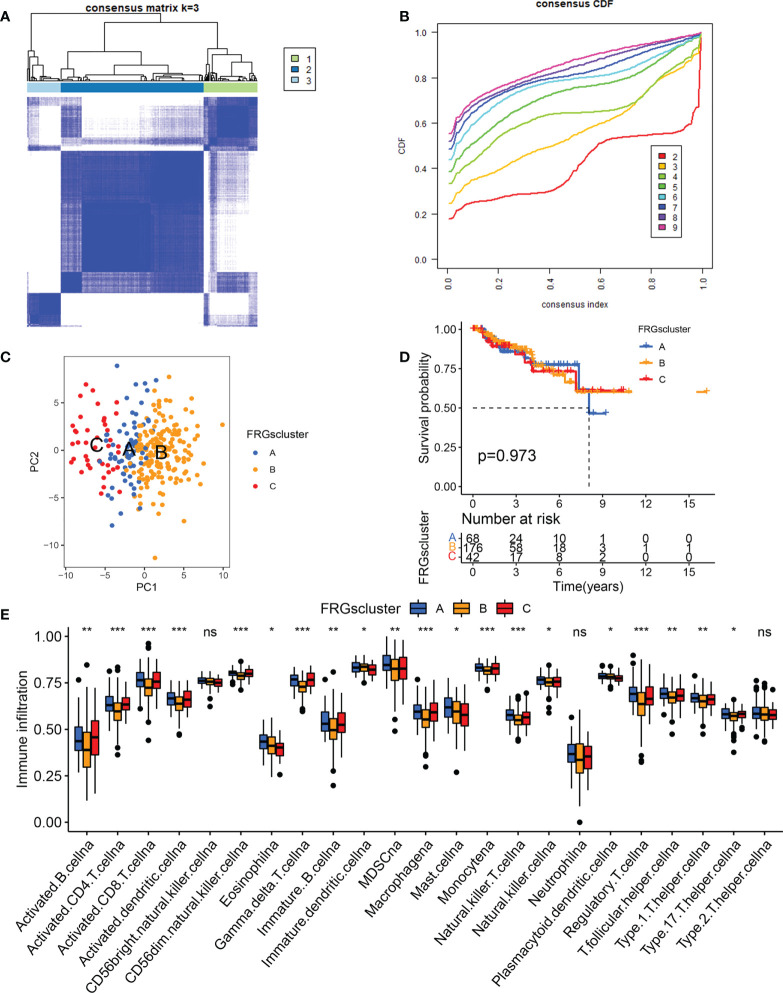
Tumor classification based on the differentially-expressed FRGs. **(A)** The consensus score matrix of all samples when k = 3 in TCGA cohorts. **(B)** Area under the curve for k = 2–9. **(C)** Principal coordinate analysis of three KIRP clusters. **(D)** Survival analysis of the KIRP subgroups comprising patients in the TCGA dataset. **(E)** The expression analysis of 23 immune cells in different KIRP subgroups *p < 0.05; **p < 0.01; ***p < 0.001; n.s., not significant.

To determine if differentially expressed FRGs had an effect on KIRP subtypes, a consensus clustering analysis was done on 321 patients with KIRP. We discovered that when the clustering variable (k) = 3, the intragroup correlations were strongest and intergroup correlations were lowest from k = 2 to 9, suggesting that patients with KIRP could be divided into three clusters based on these differentially-expressed FRGs (**Figures 2A, B**). The results of the principal coordinate analysis showed that three subtypes could be significantly separated on the basis of the transcriptome profiles of the differentially-expressed FRGs (**Figure 2C**). Notably, the survival analysis revealed that there was no significant difference in the prognosis among the three ferroptosis subtypes (**Figure 2D**). To understand the differences in biological function underlying these distinct clusters mediated by FRGs, GSVA was performed on these three subtypes. We found that there was a difference in biological function between the subtypes: cluster A was mainly enriched in glutathione metabolism, henylalanine metabolism and pentose phisphate pathway (**Supplementary Figure S2A**); cluster B was associated with folate biosynthesis, tyrosime metabolism and pyruvate metabolism (**Supplementary Figure S2B**); and cluster C was significantly involved in β-alanine metabolism, peroxisome and RNA polymerase (**Supplementary Figure S2C**). We next analyzed cell infiltration into the tumor microenvironment (TME), and the three clusters showed significantly different infiltration characteristics of TME cells. The results showed that Cluster A was significantly enriched for immunocyte infiltrative activity, including activated CD8^+^ T cells, Eosinophilna, IDCs, mast cells, MDSCs, PDCs and natural killer T cells (**Figure 2E**). Thus, these findings suggested that FRGs had an important impact on the regulation of TME.

### Construction and validation of a ferroptosis-related prognostic gene model

**Figure 3:**
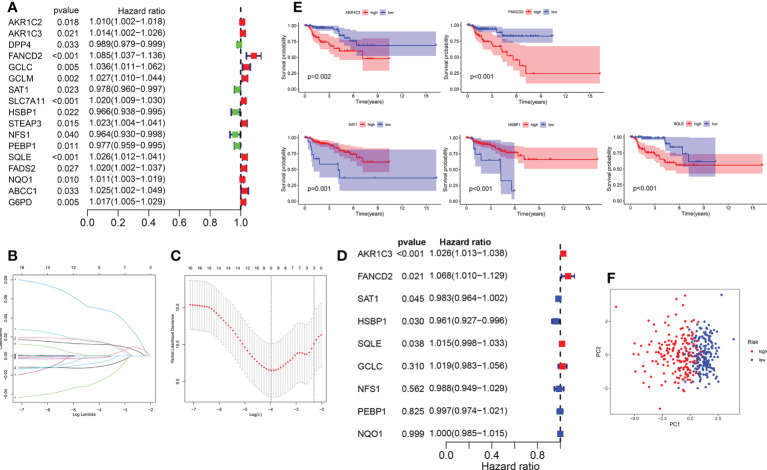
Construction of the FRGPI in training cohort. **(A)** A total of 17 prognostic related FRGs were analyzed using univariate Cox analysis. **(B)** LASSO coefficient profiles of the FRGs associated with survival of KIRP. **(C)** Plots of the cross-validation error rates. **(D)** A total of 5 independence prognostic genes were identified using multivariate Cox analysis. **(E)** Five genes were identified to be significantly associated with survival based on multivariate Cox analysis. **(F)** Principal coordinate analysis plot based on the risk score. p< 0.05 was considered statistically significant.

**Figure 4:**
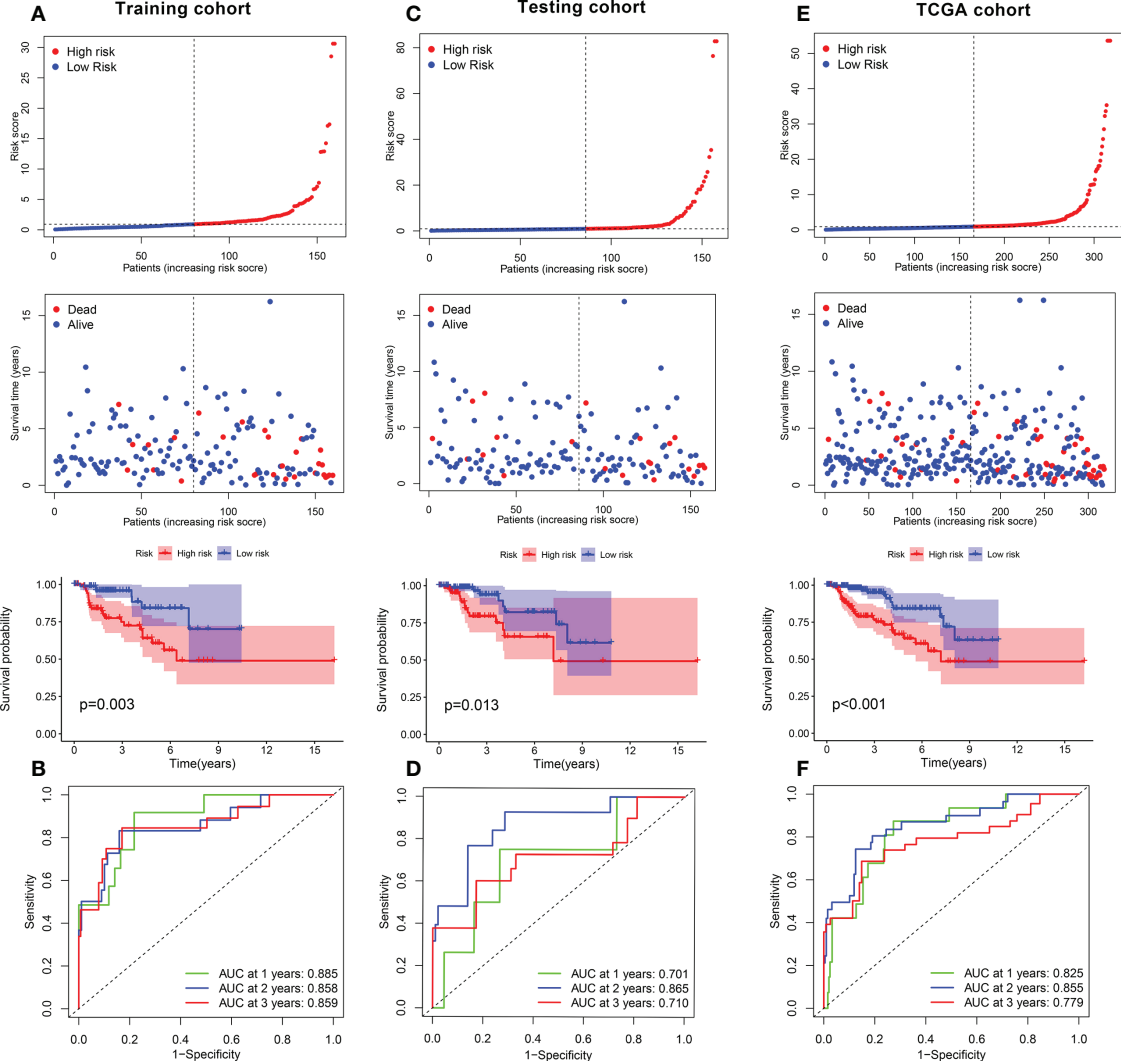
Role of FRGPI in prognosis for KIRP. **(A, C, E)** Risk score and Kaplan–Meier curve in the low- and high-risk groups comprising patients with KIRP from the training, testing and TCGA cohorts. **(B, D, F)** ROC curves to evaluate the predictive efficiency of FRGPI at 1, 2 and 3 years in the training, testing and TCGA cohorts.

The KIRP samples in the TCGA database were divided randomly into training (n = 144) and test sets (n = 142). Then, 17 prognosis-related FRGs were screened from 27 differently-expressed FRGs using the survival package (**Supplementary Figure S3**) and univariate Cox regression analyses (**Figure 3A**) in the training set; among the 17 prognosis-related FRGs, only 5 genes [aldo-keto reductase family 1 member C1 (*AKR1C3*), FA complementation group D2（*FANCD2*）, heat shock factor binding protein1 (*HSBP1*), squalene epoxidase （*SQLE*） and spermidine/spermine N1-acetyltransferase 1 (*SAT1*)]were identified the independence prognostic genes to develop a ferroptosis-related gene prognostic index (FRGPI) for KIRP patients (**Figures 3B–E**) using Lasso Cox and multivariate Cox regression analyses. The FRGPI calculated by the following formula: FRGPI (riskscore) = expression level of AKR1C3*(0.0253) + expression level of FANCD2*(0.0657) + expression level of SAT1*(-0.0173) + expression level of HSBP1*(-0.041) + expression level of SQLE*(0.0151). Patients with KIRP were stratified into the risk^hi^ and risk^lo^ groups on the biases of the median of the FRGPI (**Figure 3F**). Patients in the risk^hi^ group were associated with a poor prognosis in the training set (**Figure 4A**). And FRGPI’s AUC values were 0.885, 0.858, and 0.859 for three years, respectively (**Figure 4B**), further confirming its function as a predictive marker of KIRP. Subsequently, the testing and TCGA cohorts were utilized to confirm the prognostic utility of FRGPI, and the findings revealed that the risk^hi^ group’s survival rate was significantly lower than the risk^lo^ group’s (**Figures 4C, E**). Meanwhile, the ROC of FRGPI in the testing and TCGA cohort also showed similar results as that in the training set (**Figures 4D, F**).

### Construction of the FRGPI nomogram

**Figure 5:**
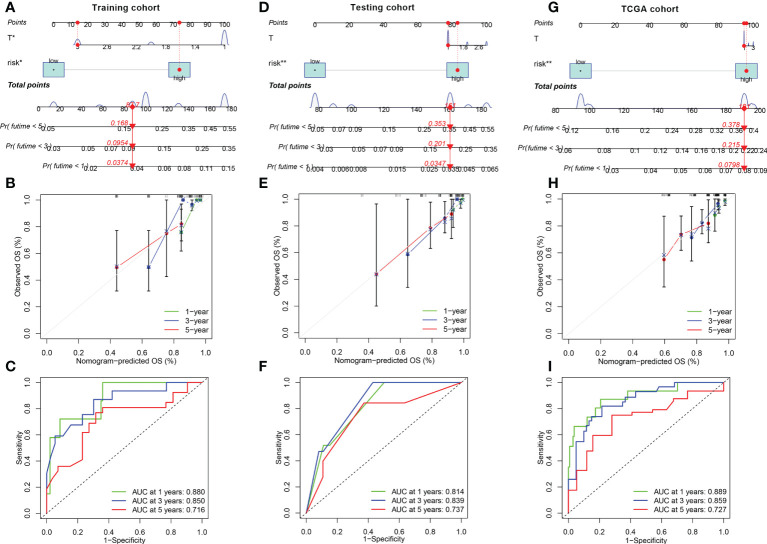
Construction and calibration of nomogram **(A, D, G)** Nomogram integrating FRGPI (risk) and other independence clinical prognostic characteristics in the training, testing and TCGA cohorts. **(B, E, H)** Calibration of the nomogram at 1-year, 3‐year and 5‐year survival in the training, testing and TCGA cohorts. **(C, F, I)** ROC curve analysis of the nomogram at 1, 2, and 3 years for KIRP patients in the training, testing and TCGA cohorts.

Next, in the training cohort, FRGPI was identified as an independent prognostic factor for patients with KIRP on the basis of univariate and multivariate Cox regression analyses (**Supplementary Figure S4A**). The sensitivity of the FRGPI was found to be higher than those of other clinical features (**Supplementary Figure S4B**), meanwhile the results was confirmed in the testing and TCGA datasets (**Supplementary Figures S3C–F**). A survival nomogram prediction model was then built based on integrate the FRGPI with other independence clinical prognostic characteristics in the training cohort. The results of the model showed that KIRP patients with high number of total points had poor prognostic (**Figure 5A**). The calibration plot displayed that an optimal agreement between observation and prediction for 1-year, 3-year and 5-year survival (**Figure 5B**). Meanwhile, the ROC of the nomogram model in predicting 1-year, 3-year and 5-year prognostic value reached up to 0.880, 0.850, and 0.716, respectively (**Figure 5C**). Besides, these results were confirmed in the testing (**Figures 5D–F**) and TCGA cohorts (**Figures 5G–I**). Therefore, these data indicated that nomogram had good accuracy in predicting the survival results of KIRP patients.

### Molecular characterization of and somatic variations in the FRGPI subgroups

**Figure 6:**
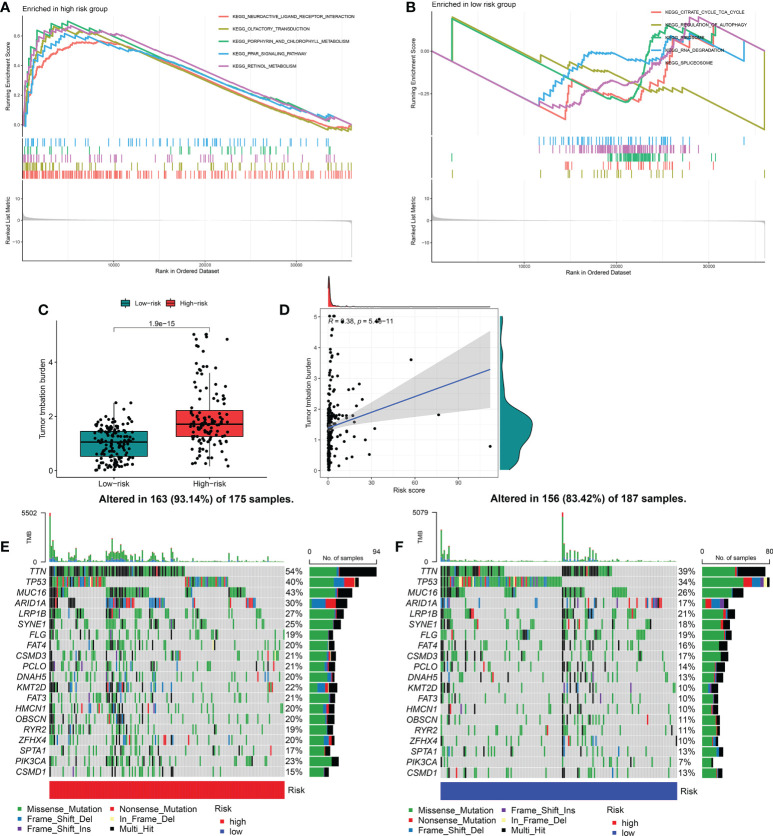
Molecular characterization and somatic variations of the distinct FRGPI subgroups. **(A)** Gene set enrichment analysis of risk^hi^ in TCGA-KIRP cohort. **(B)** Gene set enrichment analysis of risk^hi^ in TCGA-KIRP cohort. **(C)** Expression analysis of TMB in risk^hi^ and risk^lo^ groups. **(D)** Correlation analysis between TMB and risk score. **(E)** Mutational landscape of the risk^hi^ groups in TCGA-KIRP cohort. **(F)** Mutational landscape of the risk^lo^ groups in TCGA-KIRP cohort. TMB, tumour mutational burden.

To further identify the underlying differences in the biological processes among the distinct FRGPI subgroups, GSEA was performed to determine the gene sets enriched in the two FRGPI groups. As shown in **Figures 6A, B**, the top five enrichment KEGG terms in the risk^hi^ group were neuroactive ligand-receptor interaction, olfactory transduction, porphyrin and chlorophyll metabolism, PPAR signaling pathway and retinol metabolism. In contrast, the top enrichment terms in the risk^lo^ group were citrate cycle/TCA cycle, regulation of autophagy, ribosome, RNA degradation and spliceosome (detailed results of the enrichment analysis of GSEA are provided in **Supplementary Table S5**).

Tumor mutation burden (TMB) is closely related to tumor deterioration. Therefore, we further investigated the intrinsic correlation between TMB and FRGPI scores. The median TMB score was used to separate KIRP cancer samples into two groups: one with a high mutation load and one with a low mutation load. As shown in **Figures 6C, D**, the risk^hi^ group exhibited a higher TMB score than the risk^lo^ group, and there was a significantly positive correlation between the TMB and FRGPI scores. In addition, the mutation frequency of FRGs in risk^hi^ group was higher than that in risk^lo^ group (**Figures 6E, F**).

### Characteristics of TME cell infiltration and immunotherapy analysis between the two FRGPI subgroups

**Figure 7:**
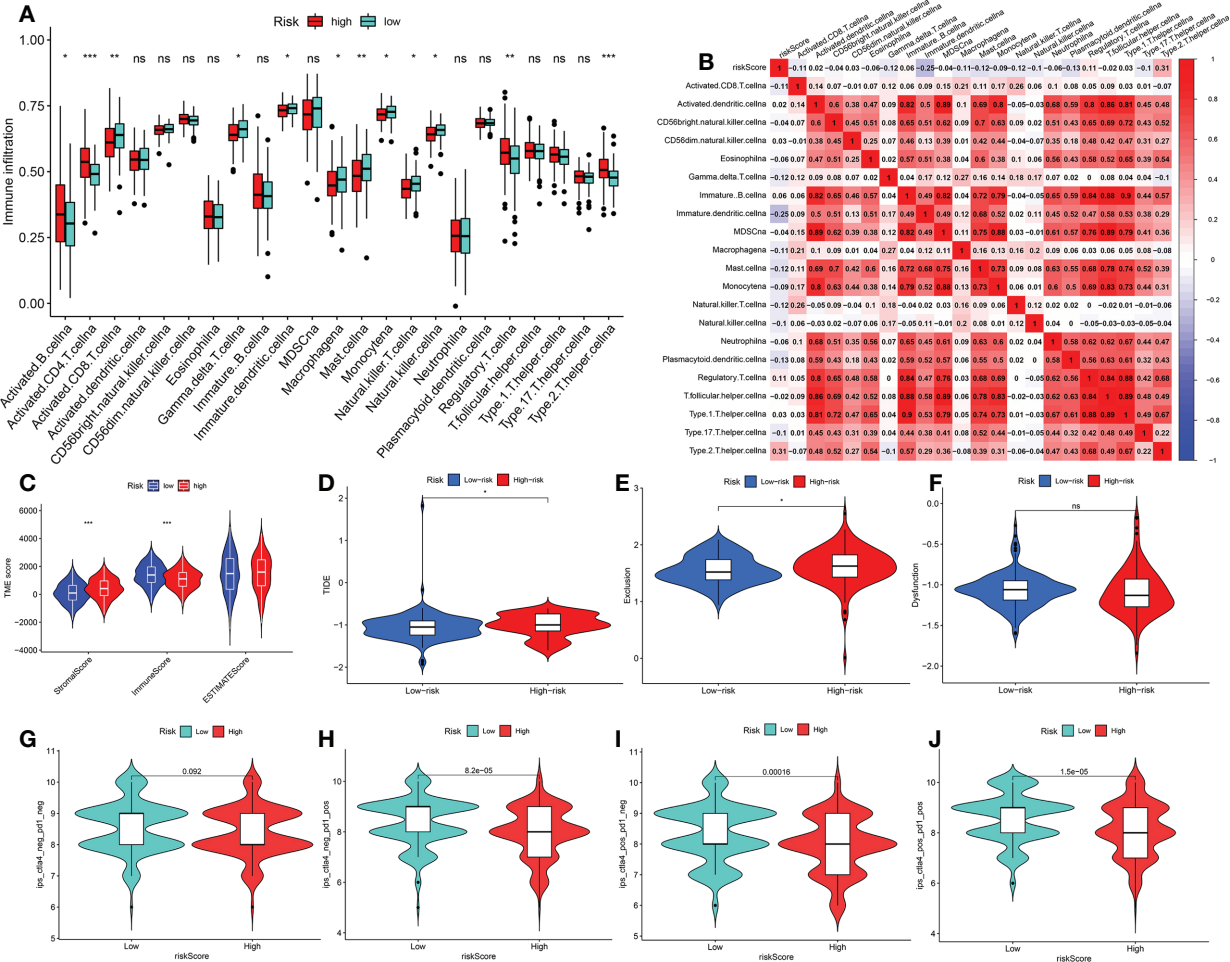
Characteristics of TME cell infiltration and immunotherapy analysis between two FRGPI subgroups **(A)** Immune landscape of 23 immune cells between risk^hi^ and risk^lo^ KIRP groups. *p < 0.05; **p < 0.01; ***p < 0.001; n.s., not significant. **(B)** Correlation analysis between 23 immune cells and risk score (FRGPI score). **(C)** The distributions of tumour tissue scores in riskhi and risklo groups **(D–F)** Comparisons of TIDE, MSI, T cell rejection and T cell dysfunction score between different FRGPI subgroups. **(G–J)** Differential analysis of response to anti-PD-L1 and CTLA-4 immunotherapy in riskhi and risklo groups. *p*< 0.05 is considered to be significant.

To compare the different immune cells in the TME between the risk^hi^ and risk^lo^ groups, the Wilcoxon test was carried out. In the risk^hi^ group, the infiltration levels of activated B cells, activated CD4^+^T cells and regulatory T cells were significantly upregulated, while the infiltration levels of activated CD8^+^T cells, macrophage, mast cells, natural killer T cells, monocyte and natural killer cells were markedly high in the risk^lo^ group (**Figure 7A**). Correlation analysis also revealed that most immune cell functions were negatively correlated with the FRGPI score (riskscore) (**Figure 7B**). In addition, the Wilcoxon test revealed that there are significant differences in the stromal, immune and ESTIMATE scores between the risk^hi^ and risk^lo^ groups in the TCGA cohort (**Figure 7C**). Immunosuppressive molecules such as PD-L1 and CTLA4 are widely used to evaluate immune response. Here, we examined the existence of many immune-related biomarkers in order to further analyze the variations in immunological activity between the FRGPI subgroups. Our analysis showed that most immunosuppressive molecules, including PD-L1 and CTLA4 and novel immune checkpoint protein (LAG3), were negatively associated with FRGPI scores (**Supplementary Figures S5A–C**). Then, TIDE algorithm was used to assess the potential clinical efficacy of immunotherapy indifferent FRGPI subgroups, the higher TIDE scores represent lower sensitive of immune therapy (31). Consistently, our result showed that the risk^hi^ group had a higher TIDE score compared with risk^lo^ group(**Figure 7D**). And the risk^hi^ group had a higher Exclusion score than risk^lo^ group (**Figure 7E**), as well as there were no noticeable differences in T-cell dysfunction scores between the risk^hi^ and risk^lo^ groups (**Figure 7F**). Meanwhile, the analysis of immunotherapy scores in the risk^hi^ and risk^lo^ groups showed that patients with KIRP in the risk^lo^ groups exhibited more significant therapeutic benefits from treatment with immune checkpoint inhibitors (ICIs) (anti-CTLA4^-^/PD-1^-^, anti-CTLA4^+^/PD-1^-^, anti-CTLA4^-^/PD-1^+^, and anti-CTLA4^+^/PD-1^+^) (**Figures 7G–J**).Therefore, these results showed that KIRP patients with risk^lo^ subtype may be more sensitive to ICIs therapy.

### Identification and validation of the high-risk genes

**Figure 8:**
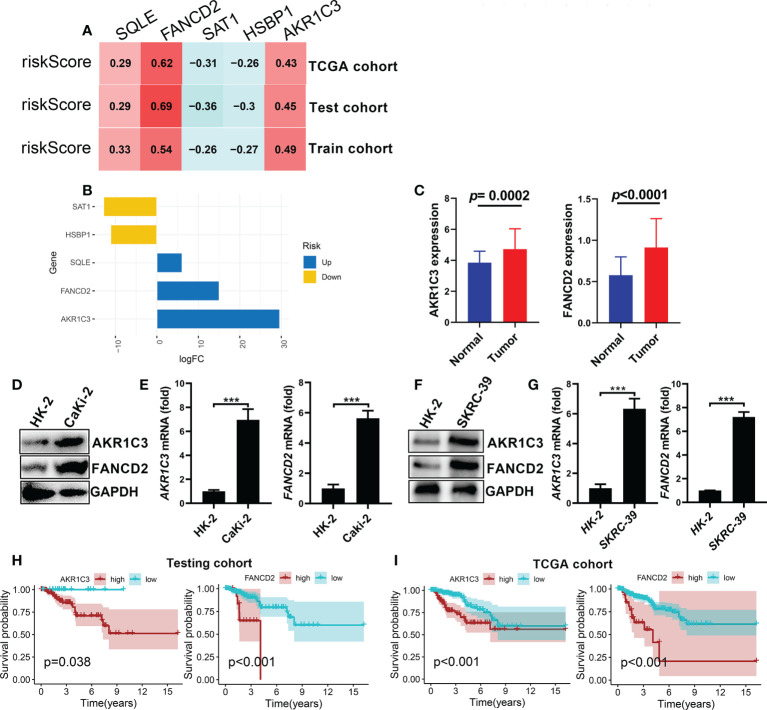
Identification and validation of the high-risk genes considered for FRGPI **(A)** Correlation analysis between the expression levels of the five genes and risk score (FRGPI score) in KIRP. **(B)** Variance multiple analysis of five cores FRGs in the distinct FRGPI subgroups (risk^hi^ and risk^lo^ groups) from the TCGA dataset. **(C)** Comparison of *AKR1C3* and *PANCD2* expression levels between KIRP and normal tissues. **(D, E)** The mRNA and protein expression levels of *AKR1C3* and *PANCD2* in human Caki-2 cells detected by q-pcR and western blotting (***p < 0.001) . **(F, G)** The mRNA and protein expression levels of *AKR1C3* and *PANCD2* in human SKRC-39 cells detected by q-pcR and western blotting (***p < 0.001). **(H, I)** Kaplan–Meier survival curves associated with *AKR1C3* and *PANCD2* in the testing and TCGA cohorts.

To further investigate the gene expression characteristics of the five genes (*AKR1C3*, *SAT1*, *FANCD2*, *HSBP1* and *SQLE*) in the FRGPI in patients with KIRP, we analysed their expression level in the risk^hi^ and risk^lo^ groups by Wilcox test. The top two high-risk genes (*AKR1C3 and FANCD2*), which were determined based on the expression patterns of these five genes in various FRGPI groups, are shown in **Figures 8A, B**. Thus, these two genes were further used to explore the role of KIRP patients. Here. As shown in **Figure 8C**, *AKR1C3 and FANCD2* expression levels were significantly higher in KIRP tissues than normal tissues. In addition, CaKi-2 and SKRC-39 KIRP cells had notably higher mRNA and protein levels of these two genes than normal human renal cells (HK-2 cells) (**Figures 8D–G**). Consistent with the previous training set analysis results, high expression of *AKR1C3* and *FANCD2* were significantly correlated with poor survival outcomes for patients with KIRP (**Figures 8H, I**). Based on the results, we inferred that *AKR1C3 and FANCD2* might play an essential role in the occurrence and development of KIRP. Then, we further performed a pan-cancer analysis of *AKR1C3 and FANCD2* across different human cancers by Wilcox test. The results showed that *AKR1C3* was mainly highly expressed in cholangiocarcinoma (CHOL), kidney renal clear cell carcinoma (KIRC), liver hepatocellular carcinoma (LIHC) and lung squamous cell carcinoma (LUSC) (**Supplementary Figure S6A**). While, *FANCD2* was highly expressed in most human cancers (**Supplementary Figure S6B**). Meanwhile, Cox regression analysis showed that the *AKR1C3* was notably related to the prognosis of adrenocortical cancer (ACC), lower grade glioma (LGG), pancreatic cancer (PAAD), prostate cancer (PRAD), thyroid cancer (THCA), head and neck cancer (HNSC), LIHC and KIRP. And *FANCD2* was markedly correlated with the prognosis of the most human cancer (**Supplementary Figures S7A, B**). Thus, these findings suggested that *FANCD2* could be served as a potential prognostic biomarker in most human cancer.

## Discussion

KIRP is a heterogeneous subtype of RCC and remains a clinical challenge due to the considerable tissue heterogeneity it exhibits, as well as its dismal prognosis and restricted therapy choices. However, the carcinogenic mechanism of KRIP is still not fully understood. Ferroptosis is a newly identified programmed cell death mechanism that differs from apoptosis and autophagy and is mainly caused by iron-dependent lipid peroxidation (32, 33). Recent research has demonstrated that ferroptosis plays a crucial role in carcinogenesis, and inducing tumour cell pyroptosis might be a viable cancer treatment (11, 34). Thus, ferroptosis is expected to be a novel promising strategy for some primary tumours. However, the role of pyroptosis and ferroptosis in malignant progression and their potential as therapeutic targets for KIRP patients is currently unknown. In this study, 41 differentially-expressed FRGs were identified by evaluating the mRNA expression levels of 57 FRGs between KIRP and normal tissues. The PPI network analyses indicated the complex interactions between these FRGs, which were markedly involved in ferroptosis, glutathione metabolism and arachidonic acid metabolism. For the first time, our study found that, based on consensus clustering analysis, these FRGs can be used to categorise patients with KIRP into two groups that exhibit significant differences in clinical and molecular features. Subsequently, an FRGPI with good prediction performance in the survival of patients with KIRP was constructed on the basis of five FRGs (*AKR1C3*, *SAT1*, *FANCD2*, *HSBP1* and *SQLE*). The FRGPI proved to be a potential prognostic biomarker for KIRP, with better survival observed in FRGPI-low patients and worse survival in FRGPI-high patients in the test, training and TCGA cohorts. Besides FRGPI showed superior prediction capacity compared to other clinical factors both in the training and validation groups.

*AKR1C3*, a crucial androgenic enzyme, plays a role in recoding the AR signal transduction in prostate cancer (35). It is also involved in the production of aromatase substrate in breast cancer (36). *SAT1* is a transcriptional target of p53 in human melanoma and lung cancer cell lines (37), and can sensitize cells to ferroptosis and inhibit tumor growth (38). Additionally, overexpressing *SAT1* was reported to lead to mitochondria-mediated apoptosis in mammalian cells (39). *FANCD2* is a protein that mediates DNA repair and inhibits ferroptosis death through transcription and transcription-independent mechanisms. Besides, *FANCD2* expression is closely related to tumorigenesis and progression (40). For example, *FANCD2* expression correlated with the activation of apoptotic and EMT pathways in clear cell renal cell carcinoma (41). *SQLE* is a key rate-limiting enzyme in the biosynthesis of cholesterol, and its overexpression was closely related to poor clinical stages and lymphatic metastasis (42). Studies have reported that SQLE is a potential prognostic marker for pancreatic cancer and has been proved to have cancer-promoting functions (43). *HSBP1* is a 76-amino-acid protein that binds to heat shock factor 1(HSF1), which can be enhanced through lin28A to regulate the stem-like characteristics of ovarian cancer (44). In this study, the mRNA expression of two core genes (*FANCD2* and *AKR1C3*) was validated in a human KIRP cell line, and the prognostic value of these two genes was confirmed in the test and TCGA cohorts; the results showed that the overexpression of *FANCD2* and *AKR1C3* was correlated with poor prognosis of patients with KIRP. Besides, FANCD2 could be a potential prognostic biomarker in different cancer types.

To investigate the underlying mechanisms, we further explored the potential biological and immune characteristics of the low-FRGPI and high-FRGPI groups; the comprehensive results showed that a high FRGPI score was closely related to the PPAR signalling pathway and retinol metabolism, while a low FRGPI score was related to RNA degradation and ribosome. It has been reported that the activation of the PPAR signalling pathway plays an important role in the development of cancer, such as colorectal cancer (45) and pancreatic cancer (46). Furthermore, ssGSEA revealed that the high-FRGPI score group had more activated B cells, activated CD4^+^T cells and regulatory T cells, while the low-FRGPI score group had activated CD8^+^T cells, macrophage, mast cells, natural killer T cells, monocyte and natural killer cells. Therefore, suppressed anti-tumour immune function in high-risk patients may be one of the reasons for their poor prognosis. These findings suggested that targeting ferroptosis could change the immune status in patients with KIRP. Notably, compared to the low-FRGPI group, we observed a higher frequency of gene mutations in the high-FRGPI group. The TIDE algorithm and immunotherapy scores indicated that patients with KIRP in the low-FRGPI groups exhibited more significant therapeutic benefits from ICIs (anti-CTLA-4 or PD-L1 immunotherapy) than those in the high-FRGPI group.

In summary, this work is the first to discover a novel signature of FRGs for predicting outcomes of patients with KIRP. This risk signature has the ability to distinguish gene mutation, functional enrichment of immune cells and molecular function-related pathways. Importantly, the FRGs *FANCD2* and *SQLE* are potential markers as well as targets for the diagnosis of KIRP. Although our study provides promising insights for a better prognosis for patients with KIRP, the study has several limitations. First, our risk model was developed and validated using only public databases; hence, prospective real-world data are required to substantiate its clinical importance. Second, more biological experiments on the high-risk genes (*AKR1C3* and *FANCD2*) and the other three genes *in vitro* and *in vivo* are required to completely understand their function in KIRP.

## Data availability statement

The original contributions presented in the study are included in the article/**Supplementary Material**. Further inquiries can be directed to the corresponding authors.

## Author contributions

HQ, CN, and XZ designed this study. HY, ML, and SL conducted the experiments and data analysis. MW, and CH prepared all the figures and tables. FQ and JN wrote the manuscript. All authors contributed to the article and approved the submitted version.

## Funding

This study was supported by grants from Guangxi Medical and Health Appropriate Technology Development and Promotion Project (#S2019018), High-Level Talent Research Projects of Youjiang Medical University for Nationalities (#yy2021sk007), and High-Level Talent Research Projects of the Affiliated Hospital of Youjiang Medical University for Nationalities (#R20196333).

## Conflict of interest

The authors declare that the research was conducted in the absence of any commercial or financial relationships that could be construed as a potential conflict of interest.

## Publisher’s note

All claims expressed in this article are solely those of the authors and do not necessarily represent those of their affiliated organizations, or those of the publisher, the editors and the reviewers. Any product that may be evaluated in this article, or claim that may be made by its manufacturer, is not guaranteed or endorsed by the publisher.

## Supplementary material

Supplementary Figure 1 PPI network and function enrichment analysis of FRGs in KIRP. **(A)** The PPI network among 41 FRGs from the STRING database. **(B)** Correlation network of the 41 differentially-expressed FRGs; red line represents positive correlation, while blue line represents negative correlation. **(C, D)** Functional annotation for FGRs using GO and KEGG enrichment analysis. KIRP, kidney renal papillary cell carcinoma; FRGs, ferroptosis-related genes; PPI, protein–protein interaction.

Supplementary Figure 2 GSVA enrichment analysis shows the activation status of biological pathways of each FRGs clusters. **(A)** FRGs cluster‐A vs. FRGs cluster‐B. **(B)** FRGs cluster‐B vs. FRGs cluster‐C. **(C)** FRGs cluster‐A vs. FRGs cluster‐C. Red represents activated pathways and blue represents repressed pathways.

Supplementary Figure 3 Survival curves of the differentially-expressed FRG in KIRP patients from training cohort. Kaplan-Meier curves of 41 differentially-expressed FRGs with LUAD patients from training cohort; *p*< 0.05 was set as the cut off for statistical significance. **Supplementary Figure S3** Independent prognostic analysis of FRGPI. **(A, C, E)** Univariate and multivariate Cox analysis of clinicopathological factors and FRGPI score (risk score) in training, testing and TCGA cohorts. **(B, D, F)** ROC curves analysis of IRGPI score and clinicopathological factors in KIRP patients from training, testing and TCGA cohorts.

Supplementary Figure 4 Correlation analyses between immune-related biomarkers and FRGPI (risk) score. **(A–C)** Correlation analysis between the expression profiles of immune related biomarkers and FRGPI scores.

Supplementary Figure 5 Expression status of *AKR1C3* and *FANCD2* in various human cancers. **(A, B)** Expression levels analysis of *AKR1C3* and *FANCD2* in various human cancers.

Supplementary Figure 6 Role of *AKR1C3* and *FANCD2* mRNA levels in prognosis for various cancers. **(A, B)** A forest plot of hazard ratios of *AKR1C3* and *FANCD2* in 33 types of cancers.
