# Supplementary material for: Ferroptosis-related gene signature predicts prognosis in kidney renal papillary cell carcinoma
Source: Front Oncol. 2022 Oct 6;12:988867. doi: 10.3389/fonc.2022.988867 (PMC9582751; doi:10.3389/fonc.2022.988867)
Supplement: Supplementary file 1 [file DataSheet_1.pdf]

SUPPLEMENTARY TABLE

Supplementary Table S1. A detail list of line cells of this study.

Cell lines	Detailed description	Source
CaKi-2 cells	Human renal papillary cell carcinoma cell line	Vinhaket corporation
CKRC-39 cells	Human renal papillary cell carcinoma cell line	Vinhaket corporation
HK-2 cells	normal human renal cells	Vinhaket corporation

Supplementary Table S2. Primer sequences of cell line samples for qPCR.

Gene Name	Forward Primer	Reverse Primer
AKR1C3	CCGAAGCAAGATTGCAGATGGC	GTGAGTTTTCCAAGGCTGGTCG
FANCD2	TTCCAGGATGCCTTCGTAGTGG	GCAGGAGGTTTATGGCAATCCC

Supplementary Table S3. Top 15 significantly enriched GO terms of the FRGs.

ONTOLOGY	Description	GeneRatio	*p* -value	Count
BP	response to oxidative stress	0.372093023	1.21E-15	16
BP	cellular response to chemical stress	0.279069767	8.85E-12	12
BP	cellular response to oxidative stress	0.255813953	3.50E-11	11
BP	fatty acid metabolic process	0.255813953	8.81E-10	11
BP	carboxylic acid biosynthetic process	0.209302326	3.20E-08	9
BP	organic acid biosynthetic process	0.209302326	3.38E-08	9
BP	sulfur compound metabolic process	0.209302326	6.17E-08	9
BP	response to metal ion	0.209302326	1.39E-07	9
BP	response to nutrient levels	0.209302326	1.04E-06	9
BP	long-chain fatty acid metabolic process	0.186046512	1.55E-10	8
BP	unsaturated fatty acid metabolic process	0.186046512	2.06E-10	8
BP	icosanoid metabolic process	0.186046512	3.30E-10	8
BP	transition metal ion homeostasis	0.186046512	8.28E-10	8
BP	olefinic compound metabolic process	0.186046512	1.88E-09	8
BP	fatty acid biosynthetic process	0.186046512	3.11E-09	8
CC	autolysosome	0.046511628	0.000256651	2
CC	secondary lysosome	0.046511628	0.000629331	2
CC	lamellipodium membrane	0.046511628	0.001061501	2
CC	organelle outer membrane	0.093023256	0.001607287	4
CC	outer membrane	0.093023256	0.001659028	4
CC	mitochondrial inner membrane	0.023255814	1.02E-21	1
CC	mitochondrial matrix	0.023255814	2.08E-20	1
CC	focal adhesion	0.023255814	2.17E-15	1
CC	cell-substrate junction	0.023255814	4.28E-15	1
CC	polysomal ribosome	0.023255814	2.62E-14	1
CC	polysome	0.023255814	2.96E-14	1
CC	organellar ribosome	0.023255814	4.56E-13	1
CC	mitochondrial ribosome	0.023255814	4.56E-13	1
CC	ficolin-1-rich granule	0.023255814	1.03E-12	1
CC	ficolin-1-rich granule lumen	0.023255814	3.94E-12	1
MF	acting on paired donors, with incorporation	0.162790698	1.25E-07	7
MF	carboxylic acid binding	0.139534884	3.23E-06	6
MF	oxidoreductase activity, acting on NAD(P)H	0.11627907	1.88E-06	5
MF	monooxygenase activity	0.11627907	3.89E-06	5
MF	oxidoreductase activity	0.11627907	1.01E-05	5
MF	oxidoreductase activity, acting on CH-OH group of donors	0.11627907	1.59E-05	5
MF	ligase activity	0.11627907	4.02E-05	5
MF	incorporation of one atom of oxygen	0.093023256	3.01E-06	4
MF	oxidoreductase activity, acting on NAD(P)H	0.093023256	9.40E-06	4
MF	lyase activity	0.093023256	0.00112069	4
MF	bile acid binding	0.069767442	1.95E-06	3
MF	alditol:NADP+ 1-oxidoreductase activity	0.069767442	2.59E-06	3
MF	17-beta-hydroxysteroid dehydrogenase (NADP+) activity	0.069767442	3.36E-06	3
MF	17-beta-hydroxysteroid dehydrogenase (NAD+) activity	0.069767442	4.27E-06	3
MF	estradiol 17-beta-dehydrogenase activity	0.069767442	9.52E-06	3

Supplementary Table S4. Enriched KEGG pathways associated with the FRGs.

ONCOLOGY	Term	GeneRatio	*p* -value	Count
KEGG	Ferroptosis	0.282051282	1.86E-17	11
KEGG	Glutathione metabolism	0.128205128	7.36E-06	5
KEGG	Arachidonic acid metabolism	0.102564103	0.00019279	4
KEGG	Chemical carcinogenesis - reactive oxygen species	0.153846154	0.000604065	6
KEGG	Cysteine and methionine metabolism	0.076923077	0.001805264	3
KEGG	Ovarian steroidogenesis	0.076923077	0.001805264	3
KEGG	Fatty acid metabolism	0.076923077	0.002486728	3
KEGG	Mineral absorption	0.076923077	0.002879624	3
KEGG	Steroid hormone biosynthesis	0.076923077	0.003018598	3
KEGG	Fatty acid biosynthesis	0.051282051	0.003259027	2
KEGG	MicroRNAs in cancer	0.153846154	0.003275604	6
KEGG	2-Oxocarboxylic acid metabolism	0.051282051	0.003631456	2
KEGG	Steroid biosynthesis	0.051282051	0.004022784	2
KEGG	Fluid shear stress and atherosclerosis	0.102564103	0.004209993	4
KEGG	p53 signaling pathway	0.076923077	0.00501434	3

Supplementary Table S5. GSEA analysis between the different FRGPI subgroups.

**ID**	**setSize**	**enrichmentScore**	**NES**	***p*- value**
KEGG_NEUROACTIVE_LIGAND_RECEPTOR_INTERACTION	223	0.566273222	1.506112395	8.79E-05
KEGG_OLFACTORY_TRANSDUCTION	67	0.66369931	1.669922251	0.000368484
KEGG_RETINOL_METABOLISM	56	0.669100508	1.669686715	0.001154093
KEGG_PORPHYRIN_AND_CHLOROPHYLL_METABOLISM	37	0.699635742	1.678922357	0.001446634
KEGG_PPAR_SIGNALING_PATHWAY	66	0.616566583	1.550574396	0.002751302
KEGG_GRAFT_VERSUS_HOST_DISEASE	37	0.664743407	1.5951909	0.004683373
KEGG_STEROID_HORMONE_BIOSYNTHESIS	49	0.633694039	1.559324927	0.004927711
KEGG_METABOLISM_OF_XENOBIOTICS_BY_CYTOCHROME_P450	62	0.611078017	1.532919924	0.005372906
KEGG_ASCORBATE_AND_ALDARATE_METABOLISM	23	0.723172899	1.642016015	0.005756296
KEGG_DRUG_METABOLISM_CYTOCHROME_P450	63	0.603261437	1.515355652	0.007388868
KEGG_CALCIUM_SIGNALING_PATHWAY	166	0.51120045	1.353714645	0.007751362
KEGG_PENTOSE_AND_GLUCURONATE_INTERCONVERSIONS	26	0.699910926	1.610124275	0.009991265
KEGG_DRUG_METABOLISM_OTHER_ENZYMES	47	0.620050159	1.521043139	0.012346251
KEGG_STARCH_AND_SUCROSE_METABOLISM	45	0.597604592	1.454384046	0.017524155
KEGG_LINOLEIC_ACID_METABOLISM	26	0.640756279	1.474040767	0.031358885
KEGG_RNA_DEGRADATION	53	-0.322757051	-1.098724849	0.023859766
KEGG_RIBOSOME	88	-0.299962245	-1.168605547	0.019285711
KEGG_REGULATION_OF_AUTOPHAGY	22	-0.460594018	-1.338492433	0.037586483
KEGG_CITRATE_CYCLE_TCA_CYCLE	30	-0.401253918	-1.256270606	0.011191541
KEGG_SPLICEOSOME	125	-0.334996697	-1.178446787	0.020832346
KEGG_LYSINE_DEGRADATION	39	-0.315611299	-1.056088718	0.043333333
KEGG_RNA_POLYMERASE	28	-0.292241116	-0.907715123	0.046076923
KEGG_PROPANOATE_METABOLISM	32	-0.223011796	-0.709549367	0.049322034

Supplementary Table S6. A table of abbreviations in this study.

Abbreviations	Description
TCGA	The Cancer Genome Atlas
PPI	protein–protein interaction
ICIs	immune checkpoint inhibitors
GO	Gene Ontology
KEGG	Kyoto Encyclopaedia of Genes and Genomes
GSEA	gene set enrichment analysis
TIDE	the tumor immune dysfunction and exclusion
ROC	the receiver operating characteristic
AUC	the area under the curve
CNVs	the mutations and copy number variations
RCC	Renal cell carcinoma
KIRP	Kidney renal papillary cell carcinoma
FRGPI	ferroptosis-related gene prognostic index
FRGs	Ferroptosis-related genes
DEGs	differentially expressed genes
Qpcr	quantative PCR
TME	Tumor microenvironment
TMB	Tumour mutation burden
AKR1C3	aldo-keto reductase family 1 member C3
SAT1	spermidine/spermine N1-acetyltransferase 1
FANCD2	FA complementation group D2
HSBP1	heat shock factor binding protein 1
SQLE	squalene epoxidase
ACC	adrenocortical cancer
LGG	lower grade glioma
PAAD	pancreatic cancer
PRAD	prostate cancer
THCA	thyroid cancer
HNSC	head and neck cancer
CHOL	cholangiocarcinoma
LIHC	hepatocellular carcinoma
LUSC	lung squamous cell carcinoma
