# Supplementary figures and images for: Ferroptosis-related gene signature predicts prognosis in kidney renal papillary cell carcinoma
Source: Front Oncol. 2022 Oct 6;12:988867. doi: 10.3389/fonc.2022.988867 (PMC9582751; doi:10.3389/fonc.2022.988867)

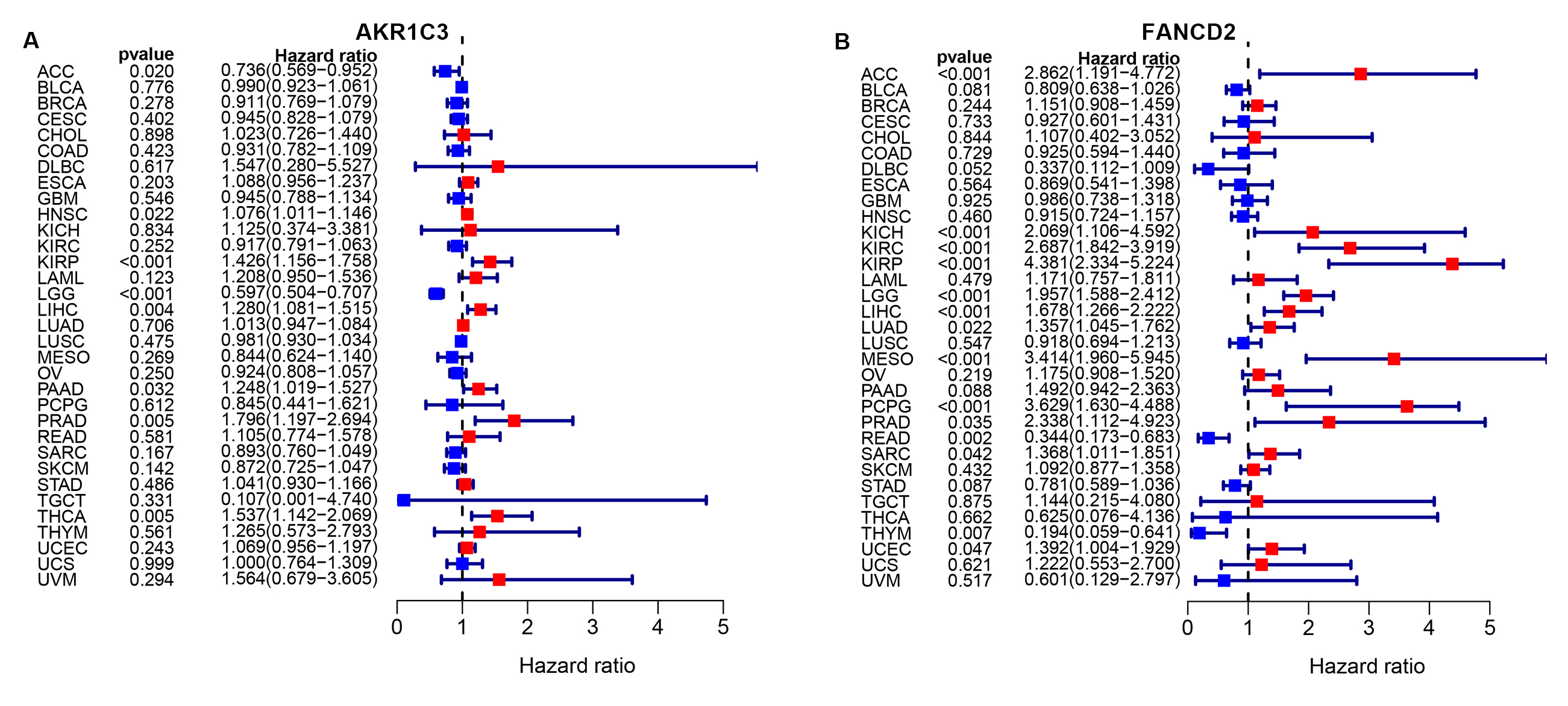

Supplement: Supplementary file 2 [file Image_7.jpeg]

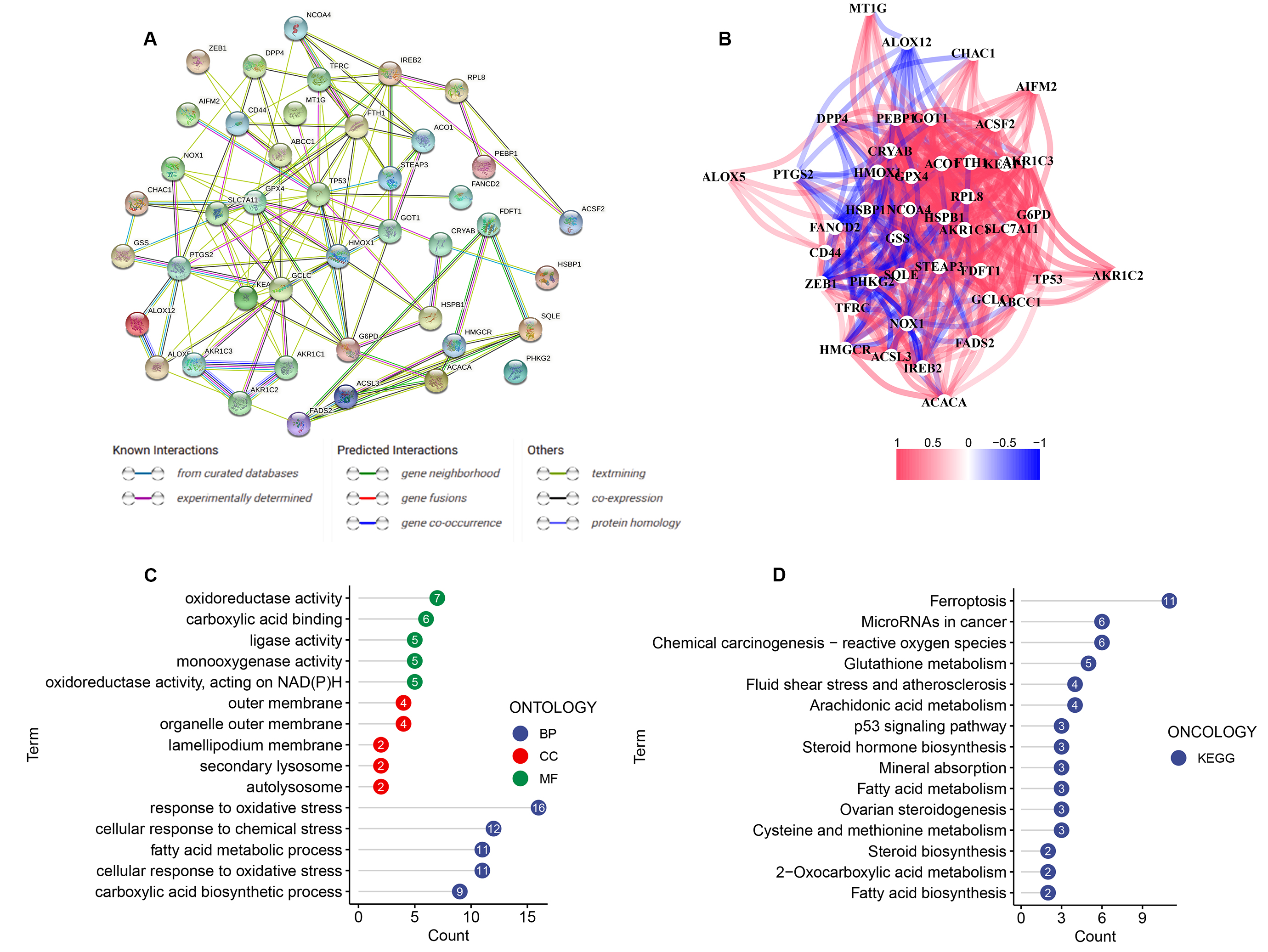

Supplement: Supplementary Figure 1 — PPI network and function enrichment analysis of FRGs in KIRP. (A) The PPI network among 41 FRGs from the STRING database. (B) Correlation network of the 41 differentially-expressed FRGs; red line represents positive correlation, while blue line represents negative correlation. (C, D) Functional annotation for FGRs using GO and KEGG enrichment analysis. KIRP, kidney renal papillary cell carcinoma; FRGs, ferroptosis-related genes; PPI, protein–protein interaction. [file Image_1.jpeg]

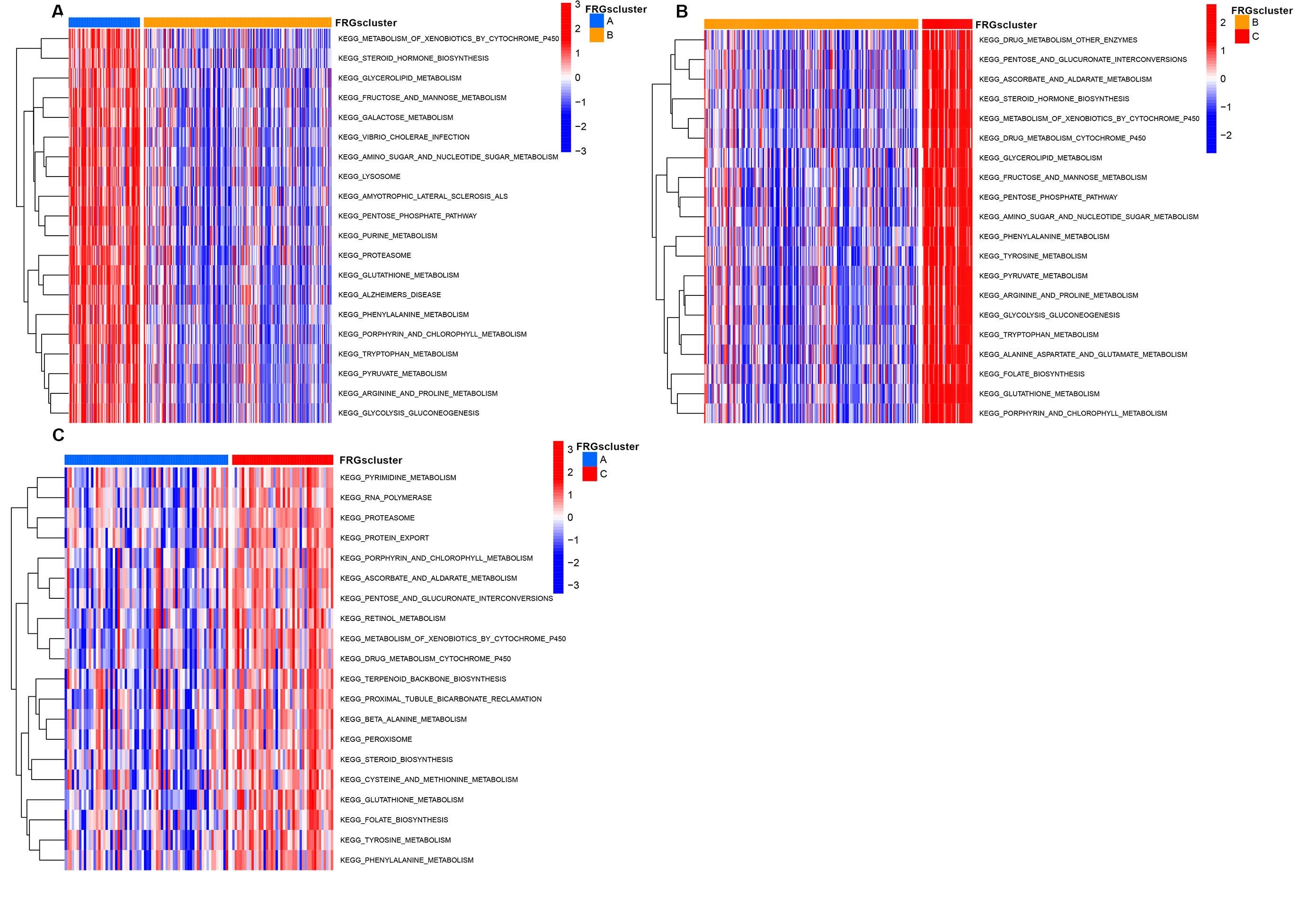

Supplement: Supplementary Figure 2 — GSVA enrichment analysis shows the activation status of biological pathways of each FRGs clusters. (A) FRGs cluster‐A vs. FRGs cluster‐B. (B) FRGs cluster‐B vs. FRGs cluster‐C. (C) FRGs cluster‐A vs. FRGs cluster‐C. Red represents activated pathways and blue represents repressed pathways. [file Image_2.jpeg]

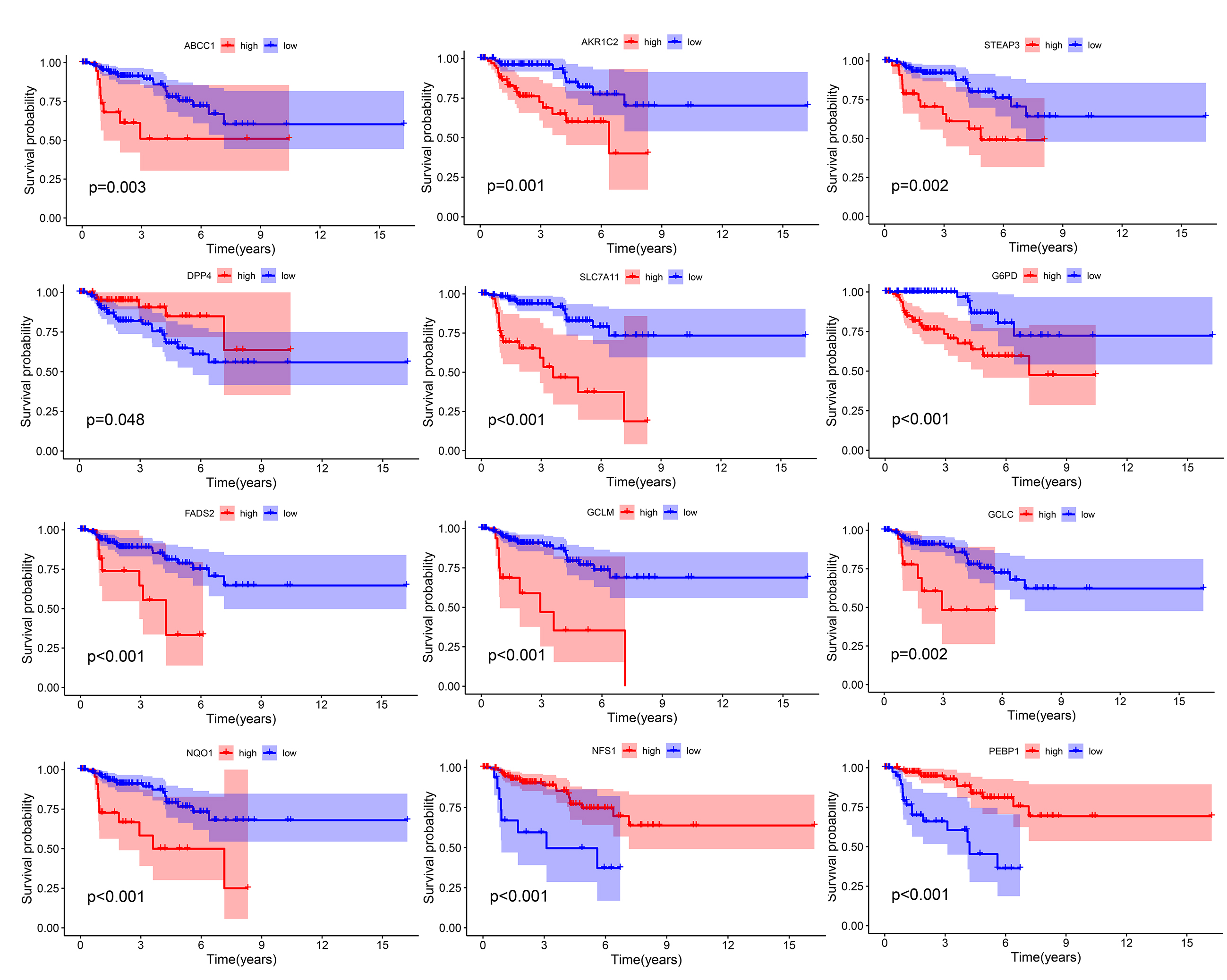

Supplement: Supplementary Figure 3 — Survival curves of the differentially-expressed FRG in KIRP patients from training cohort. Kaplan-Meier curves of 41 differentially-expressed FRGs with LUAD patients from training cohort; p< 0.05 was set as the cut off for statistical significance. Supplementary Figure S3 Independent prognostic analysis of FRGPI. (A, C, E) Univariate and multivariate Cox analysis of clinicopathological factors and FRGPI score (risk score) in training, testing and TCGA cohorts. (B, D, F) ROC curves analysis of IRGPI score and clinicopathological factors in KIRP patients from training, testing and TCGA cohorts. [file Image_3.jpeg]

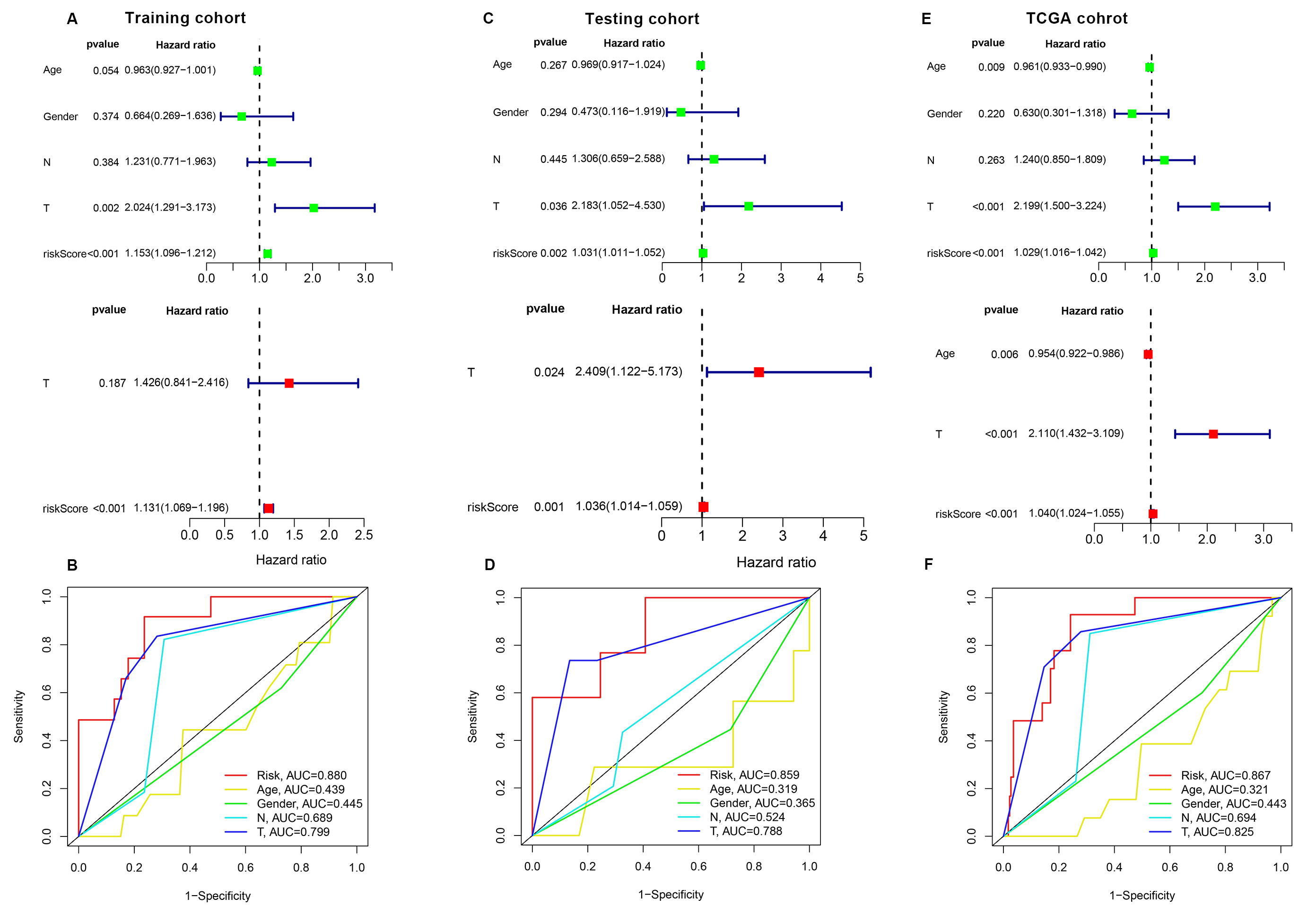

Supplement: Supplementary Figure 4 — Correlation analyses between immune-related biomarkers and FRGPI (risk) score. (A–C) Correlation analysis between the expression profiles of immune related biomarkers and FRGPI scores. [file Image_4.jpeg]

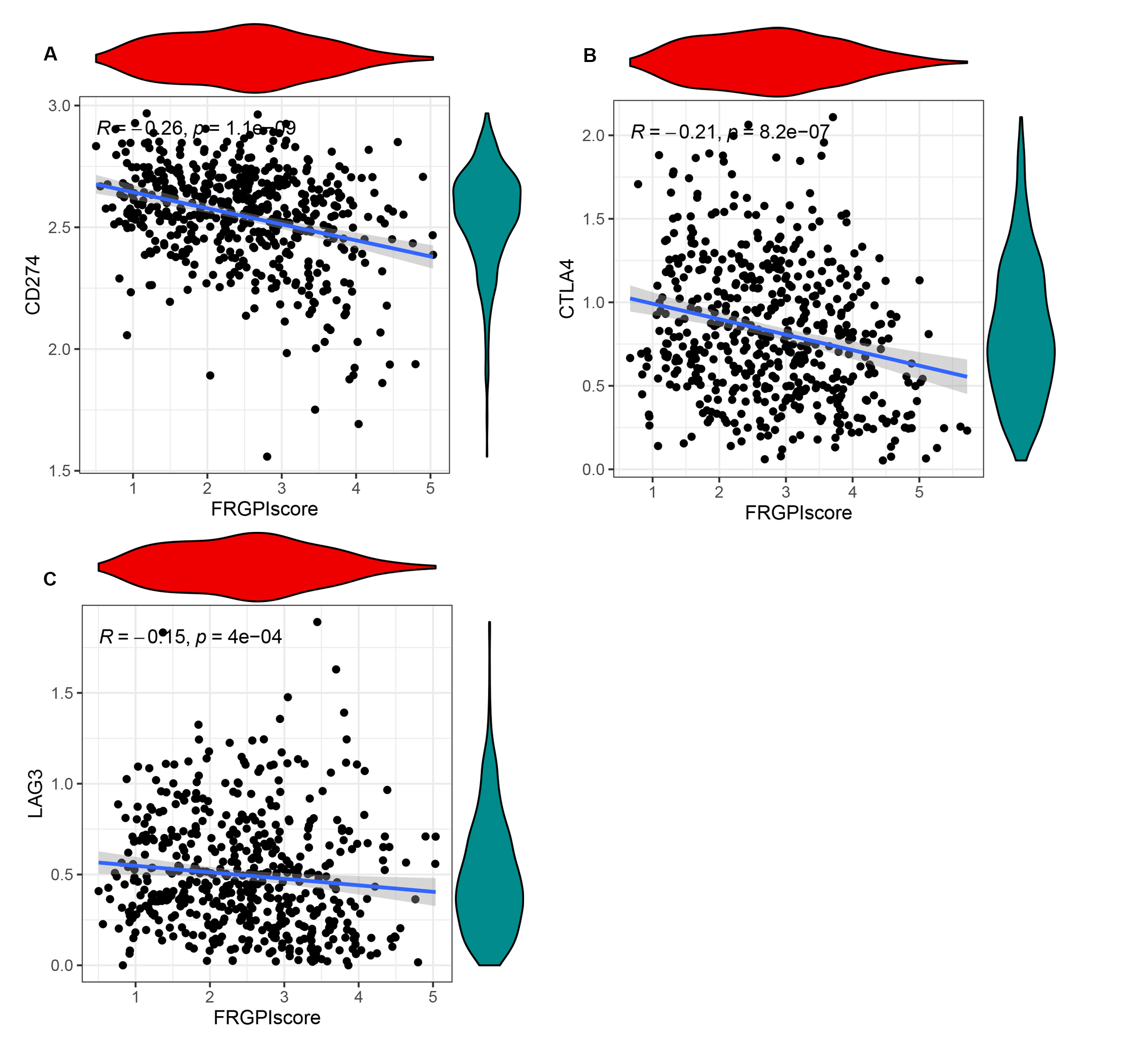

Supplement: Supplementary Figure 5 — Expression status of AKR1C3 and FANCD2 in various human cancers. (A, B) Expression levels analysis of AKR1C3 and FANCD2 in various human cancers. [file Image_5.jpeg]

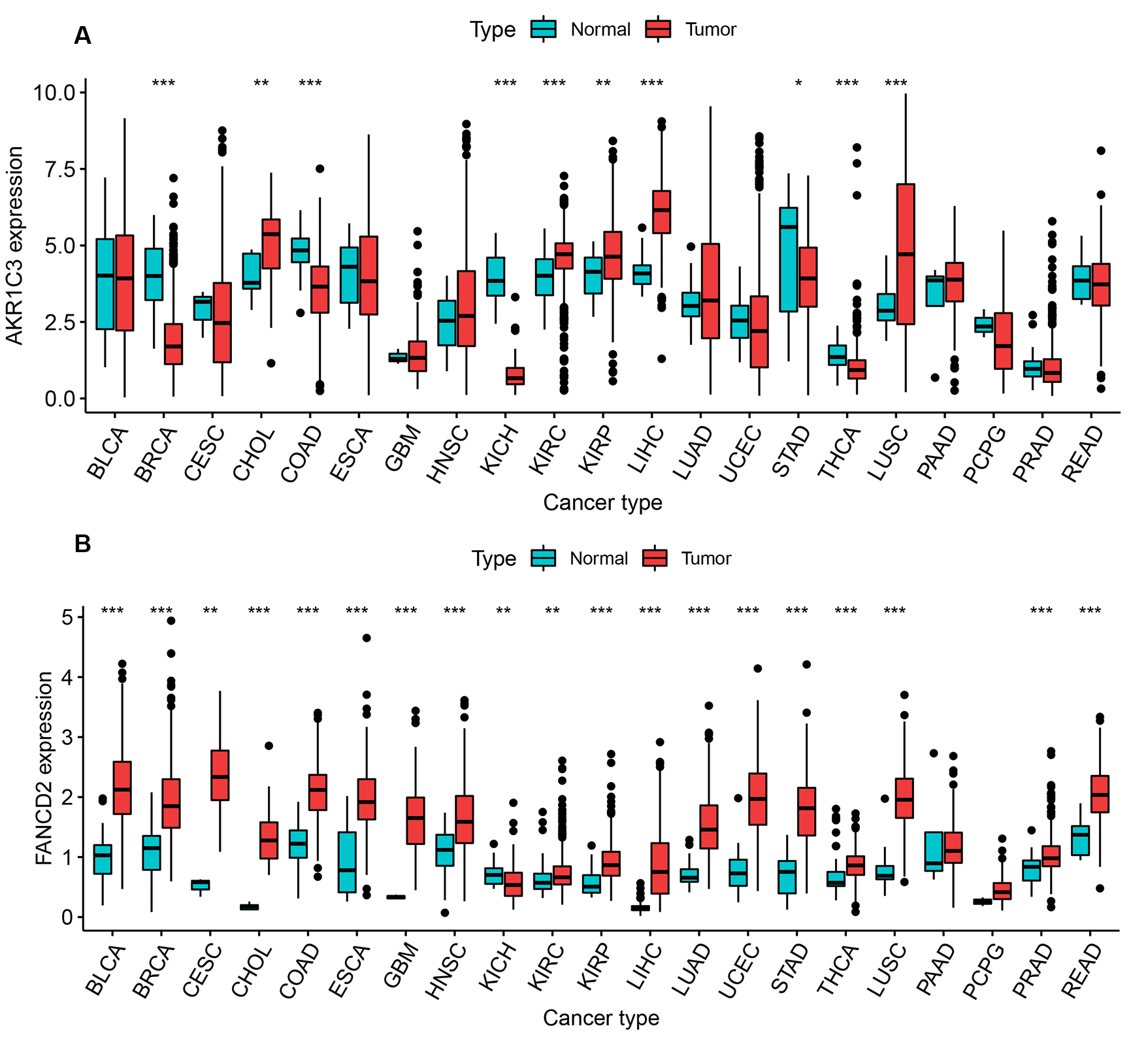

Supplement: Supplementary Figure 6 — Role of AKR1C3 and FANCD2 mRNA levels in prognosis for various cancers. (A, B) A forest plot of hazard ratios of AKR1C3 and FANCD2 in 33 types of cancers. [file Image_6.jpeg]
